# Patterns of breakdown of reading and spelling in a non‐alphabetic language: a study in Bengali

**DOI:** 10.1002/alz70857_106107

**Published:** 2025-12-26

**Authors:** Amitabha Ghosh, Arpita Roy Choudhury, Ranita Nandi

**Affiliations:** ^1^ Apollo Multispeciality Hospitals, Kolkata, West Bengal, India

## Abstract

**Background:**

Scripts of different languages differ in the representation of symbols. Abugidas, used across India and other southeast Asian countries, originate from ancient Brahmi. Here, unlike in alphabetic scripts, each symbol (“*akshara”*) represents a syllable but can also be teased apart into individual phonemes. Despite their wide reach, how increasing cognitive impairment affects reading and spelling in abugidas remains unexplored.

**Method:**

Sixty subjects (thirty each with Alzheimer's clinical syndrome‐dementia (ACS‐dem) and amnestic mild cognitive impairment (aMCI)), and 60 age‐ and education‐matched cognitively unimpaired (CU) subjects were asked to read a word list in Bengali, an abugida, and later spell the same words to dictation. The list included words containing the following: no consonant clusters; conjunct consonants where both original consonants retain their shape; one consonant in the conjunct loses its shape; both consonants in the conjunct lose their shape; plausible nonwords; irregular words. We focused on errors in reading or spelling conjunct consonants at the word level. Non‐parametric statistical analyses were performed.

**Result:**

Mean age and education of subjects were 70 (1.35) years and 14.5 (1.25) years, respectively. Both aMCI and ACS‐dem subjects read non‐words significantly worse than CU subjects (*p* <0.05) but did not differ from each other. ACS‐dem subjects were worse in reading irregular words compared to the other groups. Spelling performance in all tested parameters were worse in aMCI and ACS‐dem compared to CU (*p* <0.001) but irregular word spelling in aMCI did not differ from CU. ACS‐dem subjects spelt significantly worse than aMCI subjects on words with no consonant clusters, words where both consonants in the conjunct retained their original shapes and non‐words (*p* <0.05 in all). However, the two groups performed similarly when even one consonant in the conjunct lost its original shape.

**Conclusion:**

In ACS subjects who have Bengali as their first language, difficulty in spelling conjunct consonants within a word can be seen across all complexities of consonant cluster formation, even at the MCI stage. Reading is less severely affected and restricted to irregular words and non‐words. Our work should encourage more research on how cognitive impairment affects patterns of breakdown in other non‐alphabetic languages.

